# p38MAPK plays a pivotal role in the development of acute respiratory distress syndrome

**DOI:** 10.6061/clinics/2019/e509

**Published:** 2019-08-06

**Authors:** Ying Feng, Zhicheng Fang, Boyi Liu, Xiang Zheng

**Affiliations:** I Department of Intensive Care Unit, Taihe Hospital, Hubei University of Medicine, Shiyan 442000, Hubei Province, China; II Institute of Biomedical Research, Taihe Hospital, Hubei University of Medicine, Shiyan 442000, Hubei Province, China

**Keywords:** ARDS, p38MAPK, Pathophysiology

## Abstract

Acute respiratory distress syndrome (ARDS) is a life-threatening illness characterized by a complex pathophysiology, involving not only the respiratory system but also nonpulmonary distal organs. Although advances in the management of ARDS have led to a distinct improvement in ARDS-related mortality, ARDS is still a life-threatening respiratory condition with long-term consequences. A better understanding of the pathophysiology of this condition will allow us to create a personalized treatment strategy for improving clinical outcomes. In this article, we present a general overview p38 mitogen-activated protein kinase (p38MAPK) and recent advances in understanding its functions. We consider the potential of the pharmacological targeting of p38MAPK pathways to treat ARDS.

## INTRODUCTION

Acute respiratory distress syndrome (ARDS) is acute respiratory failure characterized by progressive dyspnea and intractable hypoxemia 1. Many clinical disorders, including pneumonia, aspiration, pulmonary contusion, severe systemic infection and multiple injuries, can promote the occurrence of ARDS 2. The development of ARDS is often accompanied by high short-term mortality rates and significant long-term outcomes (especially physical and cognitive impairment) 3. Since ARDS was initially described in 1967 by Dr. Thomas L. Petty 4, various studies have been carried out to address the clinical features (epidemiology, risk factors, treatment) and pathogenesis (biomarkers, underlying mechanisms, genetic predisposition) of this syndrome 5. Additionally, several signaling molecules and pathways have been studied to expound the pathophysiological mechanisms of ARDS and guide new therapeutic treatments. However, current treatment is mainly supportive, with mechanical ventilation; therefore, clarification of the pathophysiological mechanisms of ARDS appears to be a promising area that will lead to improved outcomes for this devastating condition.

Pro- and anti-inflammatory cytokines are strongly affected by mitogen-activated protein kinase (MAPK), and p38MAPK is the most important MAPK in stress signaling 6. There are four p38MAPK isoforms, which adds to the diversity and complexity of p38MAPK signal transduction 7. In this review, we summarize the function of p38MAPK in the development of ARDS, and provide a comprehensive understanding of p38MAPK in the molecular mechanism of ARDS.

### Promotion of inflammatory mediator production

The inflammation response can help remove invasive pathogenic microorganisms, which reduces and repairs the pathological damage caused by pathogen invasion. However, excessive inflammation leads to systemic inflammatory response syndrome or even an uncontrolled inflammatory cascade. During the development of ARDS, excessive cytokine production plays a key role in the imbalance of pro- and anti-inflammatory responses, which destroys immune homeostasis and induces an inflammation cascade 8. Several studies have shown that p38MAPK, an important signaling molecule, plays an important proinflammatory role in the development of ARDS at both the transcriptional and posttranscriptional levels.

The p38MAPK signal transduction pathway can be activated by lipopolysaccharide (LPS), stress and inflammatory factors 9,10. Evidence has indicated that p38MAPK activation is crucial for the production of inflammatory cytokines 11. Among its four isoforms, p38α MAPK was first recognized for its role in regulating proinflammatory cytokines 12. p38α MAPK was then associated with the production of IL-8 in response to IL-1 and IL-6 in response to TNF-α 13,14. IL-1β and TNF-α, which are initiation factors, not only directly damage vascular endothelial cells but also activate a series of effector cells. The proinflammatory role of p38MAPK-activated protein kinase2 (MK2), the main downstream target of p38α/β, has been widely demonstrated in various reports. MK2 knockout mice survived doses of LPS that were lethal for wild-type mice and exhibited a dramatic reduction in the cytokines IL-1β, TNF-α, and IFN-γ 15.

Although the p38MAPK pathway regulates inflammatory mediators at different levels, its role in posttranscriptional regulation is also a hot topic for researchers. The mRNAs transcribed by these genes share an AU-rich element (ARE) in their 3′ untranslated regions. These AREs can shorten the mRNA half-life and block translation. Mice with a deletion in the ARE domain of the TNF-α gene were irresponsive to LPS-stimulated, p38α-mediated TNF-α translation 16. A similar phenomenon was also observed for COX-2, another inflammatory mediator 17. Therefore, posttranscriptional regulation via AREs is a general mechanism of p38MAPK-mediated gene expression. However, the exact mechanisms by which p38MAPK carries out posttranscriptional regulation are still unclear.

### Upregulation of intercellular cell adhesion molecule-1 (ICAM-1) expression through human antigen R (HuR)

ICAM-1, also named CD54, is an important adhesion molecule. During a normal inflammatory response, neutrophils bind to ICAM-1 when blood flows through the lung capillaries. This combination of events changes the adhesion of endothelial cells and their cytoskeleton and eventually allows neutrophils to migrate in the lung, which may be an important trigger of ARDS 18. Studies have shown that the expression of ICAM-1 increases sharply in the early stage of acute lung injury (ALI) and exacerbates lung injury 19. The number of neutrophils in bronchoalveolar lavage fluid (BALF) from patients with ARDS was correlated with the severity of disease and prognosis, and neutrophil chemotaxis in these patients was greater than that in normal subjects 20. p38MAPK can regulate the expression of ICAM-1 by HuR or p53.

HuR is mainly recognized as a posttranscriptional regulatory factor. After binding to AREs, HuR can influence the half-life and/or translation of mRNAs, including TNF-α, ICAM-1, COX-2 and TLR4 21,22. Recent studies have shown that MK2 can accelerate the accumulation of HuR in the cytoplasm, which in turn enhances ICAM-1 expression, thereby promoting neutrophil adhesion to the endothelium 23. Furthermore, p38MAPK-dependent p53 phosphorylation can enhance the stability, accumulation and activation of p53, which regulates ICAM-1 expression in various physiological and pathological settings in an NF-κB-independent manner 24,25.

### Mutual regulation of p38MAPK- high mobility group box 1 (HMGB1)

HMGB1, a DNA-binding protein, mainly acts as a proximal trigger of inflammatory cytokines, such as IL-1β, IL-6 and TNF-α 26. HMGB1 overexpression may increase the incidence of ARDS by activating TLR4 in the context of trauma-hemorrhagic shock 27. Both the HMGB1 inhibitor antithrombin? and HMGB1/receptor for advanced glycation end products (RAGE) signaling pathway inhibition can improve endotoxin-induced ARDS 28, 29. With the discovery of its proinflammatory effects in the later stages of disease, HMGB1 has become a research hotspot in the field of critical care medicine. Current research shows that HMGB1 and p38MAPK promote each other, which in turn aggravates the development of ARDS.

HMGB1 mediates its activities through multiple receptors, such as Toll-like receptors (TLRs) and RAGE. By binding to these receptors, HMGB1 can activate the p38MAPK stress response pathway 30,31. As mentioned above, the activation of p38MAPK significantly stimulates the production of inflammatory factors. In sepsis, trauma or other diseases, IL-1β and TNF-α can stimulate the expression of HMGB1, which significantly increases the inflammatory response 32.

The 3′ untranslated region of HMGB1 mRNA is very long and contains AREs. HuR can enhance the translation of HMGB1 and inhibit miR-1192-mediated transcriptional repression by binding to the ARE of HMGB1 33. Based on the preceding discussion, we concluded that the p38MAPK/MK2/HuR signaling pathway may affect the pathogenesis of ARDS by regulating the translation of HMGB1 and that the p38MAPK/MK2/HuR signaling axis is a promising target for ARDS treatment.

### Promoting neutrophil accumulation in the lung and prolonging the neutrophil lifespan

Neutrophils play a pivotal role in the immune system through migrating to abnormal sites, where they function in defense. Although immune responses are important to elimination the offending microorganism, they need to be controlled to avoid causing damage to the body 34. During the early stages of inflammation, the relatively long life span of neutrophils allows these cells to better develop their defense mechanisms. After the eradication of pathogens, emigrated neutrophils undergo apoptosis and are ingested by scavenger macrophages 35,36. Abnormalities in neutrophil populations or levels of neutrophil chemotaxis have been identified in inflammation 37,38, infection 39 and ARDS 40,41. Among ARDS patients, neutrophil counts were higher in BALF from nonsurvivors than in BALF from survivors, and excessive neutrophil accumulation was associated with poor clinical outcome 42,43. p38MAPK can promote the recruitment of neutrophils in the lung by regulating the expression of surface receptors. However, LPS-induced neutrophil recruitment mainly depends on p38MAPK-mediated sensing and the prioritizing of certain signals, rather than actual migration 44.

Neutrophils are special among immune cells due to their extremely high rates of constitutive apoptosis and short half-lives 45. During inflammation, the stimulation of proinflammatory signals can delay neutrophil apoptosis, which is beneficial for neutrophils to exercise their immune function. However, in inflammatory lung disease, the prolonged survival of neutrophils and neutrophil-mediated tissue damage are attributed to deregulated neutrophil apoptosis 41,46. Although some scholars believe that p38MAPK can regulate the life span of neutrophils, this conclusion is still controversial. p38MAPK can either prolong neutrophil survival through the inactivation of caspase-3 and -8 47 or generate death-promoting signals in neutrophils by reducing myeloid cell leukemia 48.

### Destruction of the barrier function of pulmonary microvascular endothelial cells (PMVECs)

The endothelial cell lining of the pulmonary circulatory system forms a semipermeable barrier between the interstitium of the lung and the blood. Due to their position, endothelial cells are the targets of a wide variety of stresses, such as inflammatory factors, chemokines, LPS, and active oxygen species. The destruction of this barrier can lead to the infiltration of fluid into the alveolus, resulting in pulmonary disease, ALI and ARDS 49,50. Excessive inflammation and endothelial cell apoptosis are observed at the early stages of ALI, and increased endothelial and pulmonary microvascular permeability is the hallmark of ALI 51. Thus, preventing apoptosis and preserving the integrity of PMVECS is a potential therapy for ALI/ARDS. HMGB1, C-peptide and silent information regulator type-1 (SIRT1) can influence the integrity of PMVECS through p38MAPK-mediated signaling pathways.

HMGB1 is a significant signaling molecule that upregulates proinflammatory cytokines and promotes the expression of adhesion molecules 52,53. Studies have shown that HMGB1 induces dose-dependent paracellular gap formation and the loss of barrier integrity through the ligation of the RAGE, resulting in p38MAPK activation. That is, the effects of HMGB1 on endothelial barrier function depend, at least in part, on the p38MAPK pathway. JNK1 participates in the regulation of cell growth, apoptosis, and inflammation 54. The upregulation of JNK1 may influence the integrity of PMVECS. Studies have shown that C-peptide plays a role in diabetes-induced microvascular dysfunction and increases the expression of JNK1 through ERK- and p38MAPK-dependent mechanisms 55,56. A recent study indicated that SIRT1 may protect against burn-induced lung damage by attenuating the apoptosis of PMVECs through p38MAPK signaling 57. Thus, p38MAPK may participate in the pathophysiological process of ARDS through regulating PMVEC apoptosis.

### Regulation of Treg/Th17 cells functions

Treg cells are a class of CD4+ T cells that can synthesize the IL-2 receptor α chain 58. Compared with other regulatory or suppressor T cells, Treg cells have their own unique immunological characteristics. A reduction in Treg cells may result in a variety of autoimmune diseases. The interaction between innate immune cells and adaptive immune cells indicated that Treg cells might affect the excessive inflammatory response, but the exact mechanism remains to be further studied. p38MAPK can affect the progression of ARDS by regulating the expression of inflammatory factors and the balance of Th17 and Treg cells.

Th17 cells are a kind of CD4+ T helper cell that produce IL-17 59. As an early promoter of T cell-induced inflammatory responses, IL-17 can amplify inflammation by stimulating the release of proinflammatory cytokines 60. p38MAPK plays a significant role in Th17 cell function through adjusting the production of IL-17 61. Although Treg cells and Th17 cells are involved in opposite immune responses 62, Treg cells may be reprogrammed and converted into Th17 cells under certain circumstances 63,64. During the early stages of ARDS, the numbers of Th17 and Treg cells increased, and the Th17/Treg ratio was negatively correlated with prognosis 62. Furthermore, p38MAPK inhibitors could ameliorate LPS-induced ARDS through regulating the balance of Treg/Th17 cells, as reported in an abstract published in the 2016 CHEST World Congress. Thus, we speculated that p38MAPK may participate in the balance of Treg and Th17 cell differentiation.

### Interaction with endothelin (ET-1)

ET-1, which carries out a series of biological activities, was originally identified in vascular endothelial cells 65. As an inflammatory mediator, ET-1 plays a vital role in a variety of lung diseases. As previously reported, plasma ET-1 is significantly elevated in ARDS patients 66,67. Increased ET-1 IS correlated with poor outcome, increased airway and pulmonary arterial pressure, the development of permeability edema, oxygenation impairment, and multiple organ failure 66,68. ET-1 also contributes to endothelial and epithelial dysfunction through proinflammatory mechanisms 69. Studies have shown that ET-1 can upregulate the expression of arterial vascular cell adhesion molecule-1 (VCAM-1) via activating neutral sphingomyelinases mediated by p38MAPK 70. Nur77, a nuclear hormone receptor, increases rapidly after exposure to LPS or other inflammatory stimuli 71. Furthermore, Nur77 can downregulate the expression of ET-1 through suppressing LPS-induced p38MAPK and NF-κB activation 72.

## CONCLUSION

In this article, we summarize the role of p38MAPK in the development and occurrence of ARDS, including its regulation of the expression and activity of the inflammatory mediators ICAM-1, HMGB1, and ET-1; neutrophil chemotaxis and apoptosis; the balance of Treg/Th17 cells; and the PMVECs apoptosis. Our findings indicate that p38MAPK activation may promote the development of ARDS. This indication suggests the potential value of p38MAPK in the prevention and treatment of ARDS.

## AUTHOR CONTRIBUTIONS

Zheng X contributed to the study design. Feng Y and Fang Z were involved in manuscript drafting. Liu B revised the manuscript. All of the authors approved the final version of the manuscript for publication.
